# Comparative Analysis of Library Preparation Approaches for SARS-CoV-2 Genome Sequencing on the Illumina MiSeq Platform

**DOI:** 10.3390/ijms24032374

**Published:** 2023-01-25

**Authors:** Anna Gladkikh, Ekaterina Klyuchnikova, Polina Pavlova, Valeriya Sbarzaglia, Nadezhda Tsyganova, Margarita Popova, Tatiana Arbuzova, Alena Sharova, Edward Ramsay, Andrei Samoilov, Vladimir Dedkov, Areg Totolian

**Affiliations:** 1 Saint Petersburg Pasteur Institute, 197101 Saint Petersburg, Russia; 2 Research Institute for Systems Biology and Medicine, 117246 Moscow, Russia; 3 Martsinovsky Institute of Medical Parasitology, Tropical and Vector Borne Diseases, Sechenov First Moscow State Medical University, 119435 Moscow, Russia

**Keywords:** COVID-19, SARS-CoV-2, amplicon-based sequencing approach, hybridization capture sequencing approach, SNP, genomic coverage

## Abstract

Severe acute respiratory syndrome coronavirus 2 (SARS-CoV-2) has been responsible for over two years of the COVID-19 pandemic and a global health emergency. Genomic surveillance plays a key role in overcoming the ongoing COVID-19 pandemic despite its relative successive waves and the continuous emergence of new variants. Many technological approaches are currently applied for the whole genome sequencing (WGS) of SARS-CoV-2. They differ in key stages of the process, and they feature some differences in genomic coverage, sequencing depth, and in the accuracy of variant-calling options. In this study, three different protocols for SARS-CoV-2 WGS library construction are compared: an amplicon-based protocol with a commercial primer panel; an amplicon-based protocol with a custom panel; and a hybridization capture protocol. Specific differences in sequencing depth and genomic coverage as well as differences in SNP number were found. The custom panel showed suitable results and a predictable output applicable for the epidemiological surveillance of SARS-CoV-2 variants.

## 1. Introduction

Severe acute respiratory syndrome coronavirus 2 (SARS-CoV-2) is the causative agent of coronavirus disease 2019 (COVID-19), first identified in Wuhan in 2019 [1]. The new infection rapidly spread and became the cause of a worldwide pandemic, resulting in profound impacts on economic and social aspects of human life. These constituted major challenges to healthcare facilities and infrastructure that continue at present [2]. Despite the relative stabilization of the epidemiological situation, it is too early to speculate about the end of the coronavirus pandemic. Most experts agree that the virus will remain among the human population, periodically causing waves of morbidity globally [3].

Conducting genomic research on the virus in the context of the ongoing pandemic is an important tool for serving public health needs [4]. Mass sequencing permits the analysis of: viral spread and variability; the emergence of new, potentially dangerous variants [5]; the ability to evade vaccines and acquire immune escape [6]; new ways to treat and prevent disease [7]; nucleotide changes in the genome that can affect virus detection using clinical diagnostic tools, such as real-time PCR; and specific antiviral strategies or designs [8], including vaccine candidates [9,10]. The World Health Organization also highlights the importance of whole genome sequencing for public health needs, including monitoring for changes in SARS-CoV-2 genetic structure along with associated metadata, such as viral spread and activity, and the analysis of circulating strain diversity, with the tracking of SARS-CoV-2 geographic distribution over time [11].

With over three years of intensive research, a significant amount of data on SARS-CoV-2 genomic structure and features have been accumulated. An unprecedented number of viral sequences have been available in the GISAID database. These include over 14.5 mln complete, or nearly-complete, SARS-CoV-2 genome sequences (https://gisaid.org/ accessed on 24 January 2023). Phylogenetic analysis makes it possible to effectively assess ongoing processes, observe changes in the virus, respond to them in a timely manner, and make objective forecasts regarding the development of the epidemiological process [12]. Thus, SARS-CoV-2 genomic sequence data are an integral part of the effort to counter the COVID-19 pandemic, and such data are of great importance for solving problems in practical healthcare.

As SARS-CoV-2 research continues to grow, biotechnology companies offer variety of solutions for whole genome sequencing. Multiple high-throughput sequencing technologies have been used for SARS-CoV-2 sequencing, including Illumina, Ion Torrent, Oxford Nanopore Technology, and DNBSeq. Despite this variety, Illumina remains the most commonly used technology for WGS [13]. Since the development of the pandemic, several protocols have been implemented, providing complete, or near-complete, SARS-CoV-2 sequence coverage. These include shotgun metagenomic approaches, target enrichment, and target whole-genome amplification by multiplex primer sets. However, large variations in performance, e.g., genomic coverage and single-nucleotide variant (SNV) detection, occur across different protocols [14]. One widely used approach for SARS-CoV-2 sequencing is the amplicon-based ARTIC protocol (https://artic.network/ncov-2019/ accessed on 15 August 2022). Due to the significant evolution of the SARS-CoV-2 genome, this protocol has undergone several updates to improve its performance.

**Figure 1:**
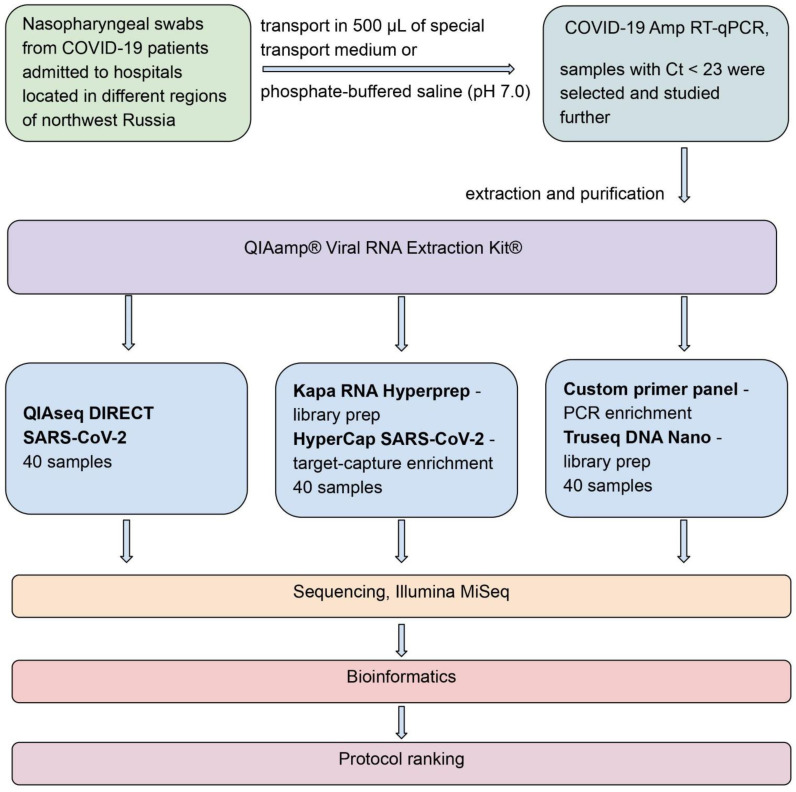
General design and experimental layout of the study. Patient nasal swabs (*N* = 40) were used to obtain RNA, which was then used to prepare WGS libraries according to three different protocols. After library preparation, each protocol’s samples were sequenced by Illumina MiSeq, followed by the bioinformatic analysis of the data.

In this study, we evaluate several approaches for library preparation for SARS-CoV-2 whole genome sequencing using the Illumina MiSeq platform. We compared commercial kits from different manufacturers in terms of data quality, genomic coverage, SNP determination, number of reads, and sequencing depth. These were: the amplicon-based QIAseq DIRECT SARS-CoV-2 Kit (Qiagen, Hilden, Germany), the target capture-based KAPA HyperCap SARS-CoV-2 Kit (Roche, Mannheim, Germany), and a whole genome sequencing custom primer panel (developed by the authors) adapted to the TruSeq Nano DNA Library Preparation Kit (Illumina, San Diego, CA, USA). The study’s general design and workflow are shown in Figure 1.

In our study, both custom and capture-based methods provided efficiently enriched SARS-CoV-2 content from clinical samples. Along with commercial kits, the custom primer panel is being successfully used for the molecular genetic monitoring of strains circulating in Russia’s Northwestern Federal District [15]. All three library preparation methods permitted the recovery of near-complete SARS-CoV-2 genomes from suitable patient samples (viral RNA load up to 23 cycles), but the sequence depth and number of raw reads varied.

## 2. Results

### 2.1. Sequencing

**Figure 2:**
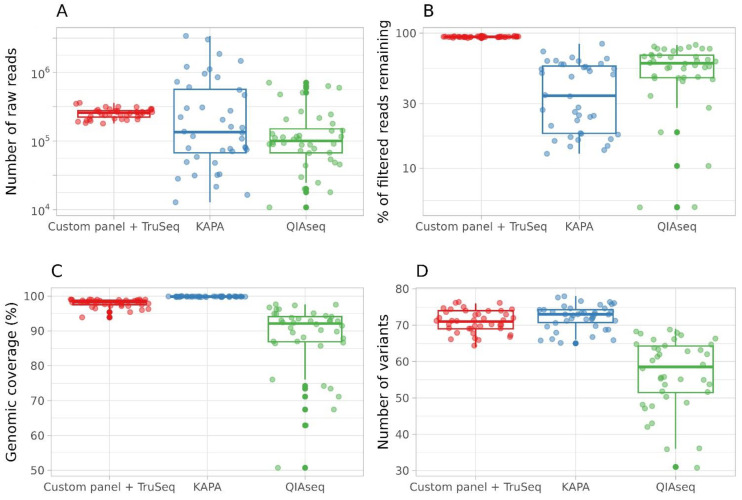
Comparison of the different library preparation kits. The *x*-axis shows the method. Boxplots show: (**A**)—number of raw reads achieved after Illumina MiSeq sequencing (*y*-axis is lg scaled); (**B**)—percent reads remaining after QC-trimming; (**C**)—percent SARS-CoV-2 genomic coverage; (**D**)—number of identified SNPs. Key: red—TruSeq DNA Nano Library Kit with custom primer panel; blue—KAPA HyperCap SARS-CoV-2; green—QIAseq DIRECT SARS-CoV-2.

After the exclusion of samples with fewer than 10,000 reads in at least one method (one TruSeq, one QIAseq, and four KAPA), forty samples were taken for analysis. The number of obtained reads varied with the KAPA and QIAseq methods; it was quite uniform with TruSeq (Figure 2A, Supplementary S2 Table S4). The parameter ‘percent reads remaining after trimming’ is presented in Supplementary S2 Table S5 and Figure 2B. The quality of data obtained with ‘custom primer panel + TruSeq DNA Nano Library Kit’ is high, whereas libraries prepared with QIAseq DIRECT SARS-CoV-2 or KAPA HyperCap SARS-CoV-2 kits yield numerous short reads.

The KAPA HyperCap SARS-CoV-2 approach offered the best genomic coverage (99.86% average genomic coverage). The ‘custom primer panel + TruSeq DNA Nano Library Kit’ approach also offered almost full genomic coverage (97.98% average coverage). Libraries prepared with QIAseq DIRECT SARS-CoV-2 resulted in the lowest and somewhat unpredictable genomic coverage, ranging from 50.71% to 97.64% (Figure 2C, Supplementary S2 Table S6). Statistical comparison with pairwise t-test showed QIAseq differed significantly from KAPA (p.adj = 5.9 × 10^−14^) and ‘custom primer panel + TruSeq DNA Nano’ (p.adj = 4.3 × 10^−11^).

**Figure 3:**
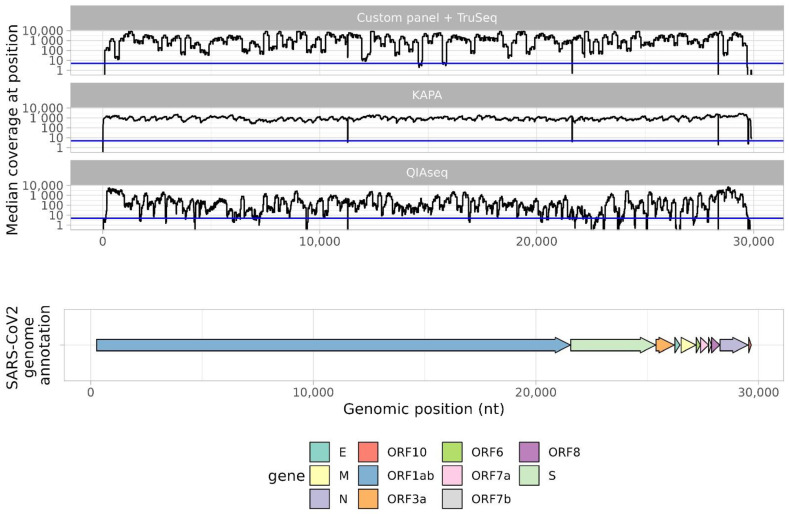
Median sequencing depth across the genome, depending on sample preparation method. The *x*-axis shows position in the SARS-CoV-2 genome and corresponding ORF. The *y*-axis shows median coverage (among 40 samples) by genomic position. The blue line represents a median coverage value of 5. A separate coverage graph is presented for each method.

Median coverage per position is shown in Figure 3. Sequencing depth from libraries prepared with KAPA HyperCap SARS-CoV-2 was the most uniform along the entire genome. The ‘custom primer panel + TruSeq DNA Nano’ and QIAseq DIRECT SARS-CoV-2 Library Kit approaches offered uneven genomic coverage in different genomic regions. The ‘custom primer panel + TruSeq DNA Nano’ approach produced quite satisfactory coverage except for several regions. With this approach, we obtained five regions without proper coverage (<5 reads in position): 14567–14706, 15682–15819, 21635–21641, 28363–28369, and 29697–29903. Only one short region (21635–21641) is located in the S gene. However, the QIAseq DIRECT SARS-CoV-2 approach produced low-sequencing depth (<5) at many positions, particularly in the S gene (21563–25384).

### 2.2. Variant Calling

**Figure 4:**
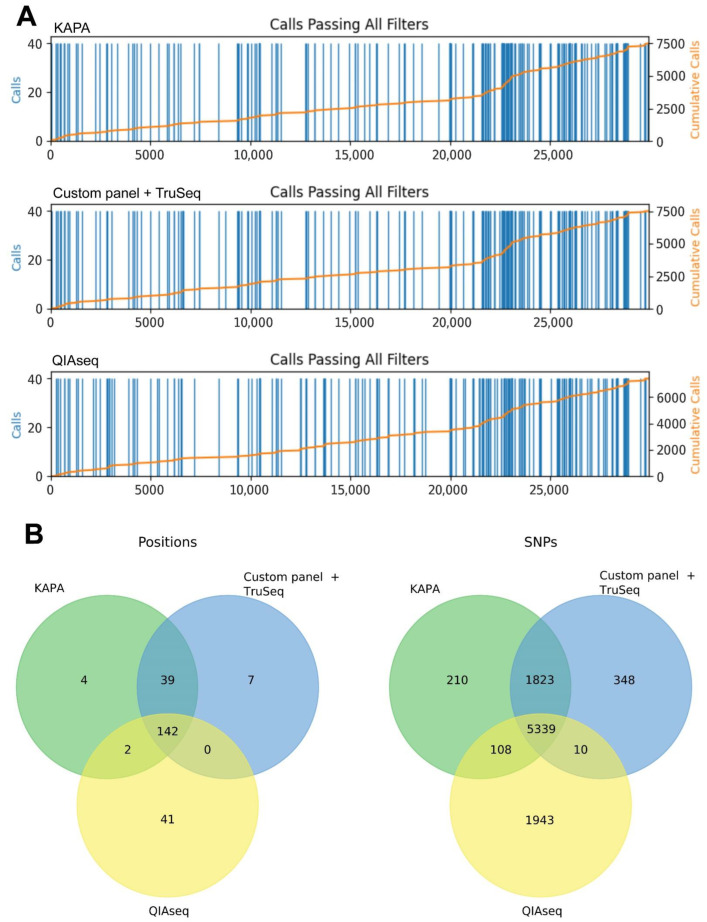
Comparison of SNP detection between different library protocols using all 40 samples. **Panel** (**A**) shows the presence of SNPs by genomic position (blue bars). The *x*-axis shows genomic position; the *y*-axis shows SNP calls. The height of the bars indicates the number of samples having calls at each position (left *y*-axis). The orange line shows the cumulative sum of variants along the length of the genome. Top—KAPA HyperCap SARS-CoV-2; middle—TruSeq DNA Nano Library Kit with custom primer panel; bottom—QIAseq DIRECT SARS-CoV-2. **Panel** (**B**) shows Venn diagrams showing the intersections of precise SNP positions (left) and the number of SNPs (right) between three methods.

All three approaches used in the study produced SNPs in the same positions subject to genomic coverage in the area. Two methods (KAPA HyperCap SARS-CoV-2 and TruSeq DNA Nano with custom panel) allowed the identification of the same number of SNPs, while less SNPs were identified with QIAseq (Figure 2D). The statistical comparison of SNP numbers was conducted with a pairwise.t.test function (R language) with the p-adjustment method “FDR”. The number of variants detected after QIAseq sequencing was significantly lower than those after KAPA HyperCap (p.adj <2 × 10^−16^) and TruSeq DNA Nano (p.adj <2 × 10^−16^). The KAPA HyperCap SARS-CoV-2 (target-capture sequencing) and TruSeq DNA Nano (amplicon-based with custom primer panel) approaches produced near-complete SARS-CoV-2 genomic sequences with few gaps. All three methods found SNPs at the same positions, and SNPs found with these approaches were all the same. The best SNP detection was seen with HyperCap. Custom panel with TruSeq DNA Nano identified equal or 1–2 fewer SNPs for each sample. QIAseq identified substantially fewer, especially in the S gene (nt 21563–25384) due to low genomic coverage (Figure 4, Supplementary S2 Table S7). Information about SNPs detected by three sequencing methods in each sample are presented in Supplementary S3 (values: 0—reference allele; 1—alternative allele; 2, 3 etc.—additional alternative alleles, if several alternatives were detected in a particular position).

Detailed statistics of sequencing, assembly, and variant calling for each sample are presented in Supplementary S1.

### 2.3. Custom Primer Panel Performance with Delta and Omicron Variants

**Figure 5:**
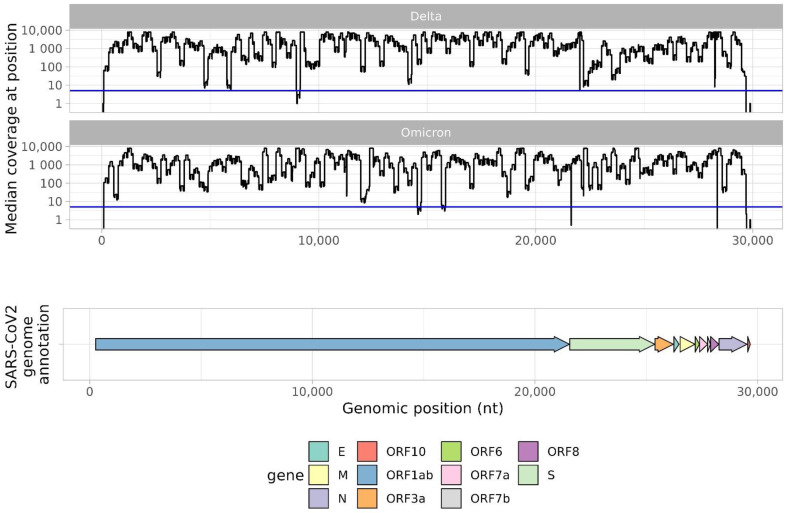
Median sequencing depth across the genome for Delta and Omicron SARS-CoV-2 samples prepared using the custom primer panel approach. The *x*-axis shows position in the SARS-CoV-2 genome. The *y*-axis shows median coverage (among 40 samples) by genomic position. The blue line represents a median coverage value of 5.

**Figure 6:**
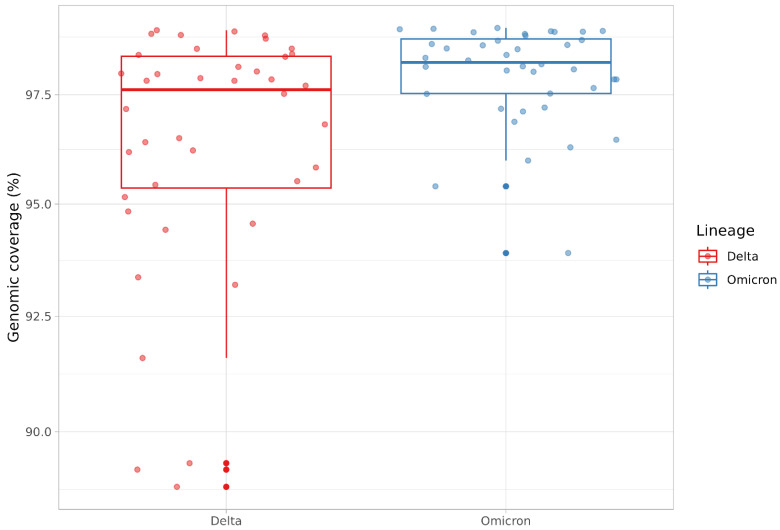
Genomic coverage for Delta and Omicron SARS-CoV-2 samples prepared with the custom primer panel approach. Boxplots shows percent genomic coverage for Delta (red) and Omicron (blue) variants.

In order to evaluate the ability of the custom primer panel to efficiently amplify the genomes of different SARS-CoV-2 variants, the assemblies of forty Delta (B.1.617.2) variants and forty Omicron (BA.1, BA.2) variants were compared. For Omicron variants, there were more regions with low-sequencing depth (Figure 5), but in general coverage per-position was still quite high, resulting in high genomic coverage. When comparing the sequencing data of the Delta and Omicron variants, there were no significant differences in genomic coverage (Figure 6). Both Delta and Omicron variants were sequenced with few gaps. Median genomic coverage was 97% or better, showing no loss of primer specificity when the SARS-CoV-2 variant changed.

## 3. Discussion

For the WGS of specific targets such as SARS-CoV-2, amplicon-based methods are well appreciated. They are the most sensitive, while avoiding DNA contamination from humans or other accompanying microorganisms. Since such protocols are less demanding in terms of the quality of source material and are quite simple to perform, they can be cost-effectively implemented in routine laboratory studies [16]. However, amplification sequencing has a number of disadvantages: polymerase errors [17,18]; uneven amplification of genomic regions associated with RNA damage; or unanticipated primer interactions [19].

Panels using multiplex RT-PCR for RNA virus sequencing are widely used. They are offered by many manufacturers or consortiums: the well-known ARTIC panel; CleanPlex^®^ SARS-CoV-2 Research and Surveillance Panel (Paragon Genomics); QIAseq SARS-CoV-2 (Qiagen); AmpliSeq SARS-CoV-2 Research Panel (Illumina); and others. The availability and cost of reagents, as well as the speed of preparatory steps and sequencing, can become essential factors determining the choice of WGS methods.

For the genetic monitoring of SARS-CoV-2 variants circulating in Russia’s Northwestern Federal District, a custom panel of primers for WGS, compatible with commercial kits, was developed [15]. The primer panel was combined with the TruSeq Nano Library Preparation Kit (Illumina) and Illumina CD Indexes, allowing near-complete SARS-CoV-2 genome sequencing on the Illumina MiSeq platform. Data obtained during the current study reflect stable and reproducible sequencing results. In the protocol, each obtained viral cDNA sample (after reverse transcription) was subjected to multiplex PCR to generate viral genome amplicons. Due to the presence of a stage with amplification from cDNA and electrophoresis, the approach allows the selection of amplicons by quality for subsequent library preparation. Thus, there is potentially less sample loss when creating libraries from enriched genomes.

The custom approach is less demanding in terms of input material quality, which is especially important for operational work with samples from clinics in different regions. The primer panel has been used to identify SARS-CoV-2 variants in various clinical specimens. It was found to be sensitive enough to identify viral RNA in patient samples with up to Ct 23 in RT-PCR, which corresponds to a viral load of N × 10^7^ particles per sample (where N may vary). We found another significant advantage to our custom panel: if necessary, having a set of primers covering the entire genome, we can refine any regions using sequencing by the Sanger method.

When comparing amplicon-based approaches, the mean genomic coverage was 88% for the QIAseq DIRECT SARS-CoV-2 Library Kit. With the custom primer panel, it was 97.98%, with better sequencing depth and read quality. The emergence of new SARS-CoV-2 variants with novel sets of mutations can reduce primer annealing efficiency, which is one of the weaknesses of amplicon-based approaches [13]. The lower sequencing depth in the S gene region (the most variable) with the QIAseq DIRECT SARS-CoV-2 primer panel, and reduced number of identified SNPs compared with other approaches, most probably is a consequence of this weakness. We used the standard kit released in 2021. Currently, Qiagen offers additional Region Booster kits.

In addition to amplicon-based methods, another common approach, capture-based sequencing, has proven itself well. Its advantages include better sequencing uniformity than amplicon-based methods [20] and fewer PCR artifacts [13]. Indeed, when comparing our results, the KAPA HyperCap SARS-CoV-2 approach showed uniform coverage and fewer gaps regardless of the number of reads, but imposes rather stringent requirements on the quality of the source material. We have found that, while the hybrid capture-based process is very labor intensive, requires more input samples, and requires intermediate quality control of libraries by capillary electrophoresis, there is often more of a need for additional purification from dimer adapters than with amplicon-based technologies. Overall, it is effective in targeting the entire genome and discovering new variants, which is in line with [21]. In our observations, it is more difficult to achieve the same sequencing quality and number of reads for each sample in the pool with this approach. Because hybridization capture is capable of handling millions of targets per panel and many overlapping data acquisition probes, this approach is best used to detect large numbers of targets [13,21,22], such as with the QIAseq xHYB Viral Respiratory Panel (Qiagen) and Respiratory Virus Oligo Panel (Illumina). Another important aspect is the difficulty of designing probes compared to the amplicon method, where it is sufficient to synthesize overlapping primers and perform PCR.

When summarizing our observations, both amplicon-based and target-capture-based approaches are suitable for SARS-CoV-2 research. Considering their pros and cons, capture-based methods are valuable for studying new mutations due to full genome coverage and uniform sequencing depth. However, the peculiarities of library preparation and the uneven distribution of readings among the samples do not allow it to be productively used for routine monitoring needs. At the same time, the amplicon-based approach has dropouts in reads in the same places and is highly dependent on PCR efficiency, which is also difficult to control when working with multiplexes. Despite this, the simplicity and reproducibility of the amplicon method, combined with a properly designed primer panel, allows us to use it for routine monitoring.

## 4. Materials and Methods

### 4.1. Clinical COVID-19 Specimens and Detection

During the routine study of SARS-CoV-2 genetic diversity in Russia, nasopharyngeal swabs from COVID-19 patients (admitted to hospitals located in different regions of northwest Russia) were collected and delivered to the Saint Petersburg Pasteur Institute for sequencing and further genetic study. Swabs were collected in 500 µL of special transport medium, or phosphate-buffered saline (pH 7.0), and stored at −20 °C until analysis.

For SARS-CoV-2 detection and to assess viral load, swabs were thoroughly analyzed using the COVID-19 Amp RT-PCR Kit (Saint Petersburg Pasteur Institute, St. Petersburg, Russia) [23], according to the manufacturer’s recommendations. For the identification of Omicron variants, a previously developed RT-PCR assay was used [15]. SARS-CoV-2-positive samples (46 Omicron and 40 Delta) featuring Ct values of 23 or less were selected and studied further.

### 4.2. RNA Isolation

Total nucleic acid samples were obtained by extraction and purification using the QIAamp^®^ Viral RNA Extraction Kit^®^ (QIAGEN, Hilden, Germany) with the QIAcube Connect automatic station (QIAGEN, Hilden, Germany), according to the manufacturer’s recommendations. RNA was eluted with 50 µL of AVE Buffer^®^ (QIAGEN, Hilden, Germany) and stored at −70 °C until molecular analysis. The quality of the template RNA was determined using a Nanodrop spectrophotometer; RNA with A260/A280 ratio of 1.8–2.2 was used for the study. The amount of isolated RNA was determined using a Qubit fluorimeter.

### 4.3. Library Preparation

#### 4.3.1. QIAseq DIRECT SARS-CoV-2 Library Kit

Libraries were prepared according to the protocol provided by the manufacturer. Briefly, 5–10 ng of purified viral RNA was used for reverse transcription reaction containing: 5 μL purified RNA; 1 μL RP Primer (random hexamer); 4 μL 5x Multimodal RT Buffer; 8 μL nuclease-free water; 1 μL RNase inhibitor; and 1 μL EZ Reverse Transcriptase. The resulting mixture was incubated in a thermal cycler (42 °C for 30 min, 85 °C for 5 min, 4 °C hold).

The target enrichment of the resulting cDNA was then performed with 2 primer pools. For this, two 2.5 μL cDNA aliquots (5 μL combined) were mixed with 2.5 μL pool (DIRECT SARS-CoV-2 Pool 1 or 2.5 μL DIRECT SARS-CoV-2 Pool 2), 12.5 μL QIAseq 2x HiFi Mix, and 8 μL nuclease-free water. PCR conditions were: heat activation 1 cycle 98 °C 2 min; 35 cycles (denaturation 98 °C 20 s, annealing/extension 63 °C 3 min); 4 °C hold. Reaction products were combined and purified on 50 µL QIAseq Beads (1:1 ratio). Of the resulting purified amplicons, 100 ng were used for PCR with 2 μL QIAseq DIRECT UDI Index and 25 μL 2x QIAseq HiFi mix. PCR conditions were: heat activation 1 cycle 98 °C 2 min; 7 cycles (denaturation 98 °C 20 s, annealing 63 °C 30 s, extension 72 °C 30 s); 4 °C hold. The reaction products were combined and purified on 50 µL of QIAseq Beads. For quality control, 10 ng of libraries were used for capillary electrophoresis. The resulting libraries were approximately 360–380 bp. All libraries were diluted to 4 nM. Five microliters of each 4 nM library were taken to produce an equimolar pool of libraries, followed by the denaturation protocol.

#### 4.3.2. Custom Primer Panel with the TruSeq Nano DNA Library Preparation Kit

In order to obtain near-complete genome sequences of SARS-CoV-2 variants (excluding 5′ and 3′ ends), a total of 138 primer pairs were designed with amplicon lengths of about 300 nt with 50 nt overlaps [15]. Purified viral RNA (5–10 ng) was used for the reverse transcription reaction with the Reverta-L Kit. For the reaction, 10 μL of purified RNA, RT-mix, RT-G-mix-1, and reverse transcriptase were mixed. The resulting mixture was incubated in a thermal cycler (35 °C, 30 min).

The target enrichment of the resulting cDNA was then performed with 6 primer pools. The reaction mixture (2 µL cDNA, 1 µL of each primer mix, 2x HiFi mix, nuclease-free water) was subjected to PCR. Thermal cycling parameters were: 95 °C for 3 min; 35 cycles (93 °C for 10 s, 57 °C for 30 s, 72 °C for 30 s); final extension at 72 °C for 5 min; 4 °C hold. Reaction products were combined and purified on AMPure Beads (1:1 ratio). The resulting purified amplicons (100 ng) diluted in RSB (Resuspension Buffer) were used for Repair Ends Reaction. After mixing 100 ng of each sample 40 μL ERP 2 reaction mixtures were heated in a thermal cycler (30 °C, 30 min).

The reaction products were then purified on 100 μL of sample purification beads (SPB). For the adenylation reaction, 12.5 μL A-Taling mix (ATL) was added to the purified samples, followed by incubation in a microheating system for reaction (37 °C for 30 min, 70 °C for 5 min, 4 °C for 5 min). The next step ligated index adapters to the ends of the DNA fragments. Adenylation reaction products were mixed with 2.5 μL Ligation Mix (LIG 2), 2.5 μL RSB, and 2.5 μL DNA Adapters. Reactions were placed on a microheating system (30 °C, 10 min). Afterwards, 5 μL Stop Ligation Buffer (STL) was added, and the reaction was incubated for 5 min.

Reaction products were then purified on SPB (1:1 ratio) in two rounds to remove unligated adapters. Afterwards, purified libraries were enriched by PCR using 5 μL PCR Primer Cocktail (PPC) and 20 μL Enhanced PCR Mix (EPM). Conditions were: heat activation 1 cycle 98 °C 2 min; 8 cycles (denaturation 98 °C 20 s, annealing 60 °C 15 s, extension 72 °C 30 s); 4 °C hold. Reaction products were then purified on SPB at a ratio of 1:1. For quality control, 10 ng of libraries were used for capillary electrophoresis; the resulting libraries were approximately 420–440 bp. All libraries were pooled in equimolar ratios into one 4 nM pool, followed by the denaturation protocol.

#### 4.3.3. KAPA HyperCap SARS-CoV-2 Library Kit

For the fragmentation and priming reaction, 5–10 ng of purified viral RNA was mixed with 10 µL of FFPE and incubated at 85 °C. After 4 min of fragmentation reaction, first-strand synthesis was carried out. A mix including 11 μL first strand synthesis buffer and 1 μL KAPA script was added to the RNA and incubated (25 °C for 10 min, 42 °C for 5 min, 70 °C for 15 min, 4 °C hold). For second-strand synthesis, 31 μL 2nd Marking Buffer and 2 μL ‘Strand Synthesis and A-tailing Enzyme Mix’ were added to the reaction mixture and incubated (16 °C for 30 min, 62 °C for 10 min, 4 °C hold).

Following second-strand synthesis, adapters were ligated. For this, 40 μL Ligation Buffer, 10 μL DNA Ligase, 25 μL nuclease-free water, and 2.5 μL adapter were added to the reaction mixture, followed by incubation for 16 h at 8 °C (these conditions reduce adapter dimer formation). After adapter ligation, libraries were cleaned with KAPA Pure Beads (1:0.8 ratio) and enriched by PCR with 25 μL HiFi HotStart ReadyMix and 5 μL Illumina Primer Mix (10x). Conditions were: heat activation 1 cycle 98 °C 45 s; 18 cycles (denaturation 98 °C 15 s, annealing 60 °C 30 s, extension 72 °C 30 s); 72 °C 1 min; 4 °C hold.

For quality control, 10 ng of cleaned libraries were used for capillary electrophoresis; the resulting libraries were approximately 240–300 bp. The libraries with unique indices were mixed in an equimolar ratio into a pool, with a total mass of 1500 ng. For hybridization, the library pool was supplemented with 20 μL COT DNA, purified with KAPA HyperPure Beads (1:1 ratio), and eluted in Universal Enhancing Oligos. The pool with KAPA HyperPure Beads in Universal Enhancing Oligos was added to the hybridization mix, including Hybridization Buffer, Hybridization Component H, and PCR-grade water. After a 2 min incubation at room temperature, the pool was placed on a magnetic stand. After the clearing of the liquid, the supernatant was transferred to a tube with 4 μL of KAPA HyperCap SARS-CoV-2 probes and incubated (95 °C for 15 min, 55 °C for 16–20 h). Then, after enrichment and washing of the hybridized pool, libraries were amplified with Kapa HiFi HotStart ReadyMix and Post-LM-PCR Oligos. Conditions were: heat activation 1 cycle 98 °C 45 s; 8 cycles (denaturation 98 °C 15 s, annealing 60 °C 30 s, extension 72 °C 30 s); 72 °C 1 min; 4 °C hold. To check the quality and size of the final library, 10 ng of the purified enriched pool was analyzed by capillary electrophoresis. Fragment distribution was in the 150–500 bp range, with a median of 320–400 bp.

The comparison between the library preparation protocols is presented in Table 1.

### 4.4. Pool Denaturation, Dilution, and Sequencing

A total of 5 microliters of the 4 nM library pool was mixed and incubated with 5 µL 0.1 N NaOH to denature the dsDNA (5 min, room temperature). PhiX library was also denatured using 0.1 N NaOH. The denatured library pool and PhiX library were each diluted to 20 pM by adding HT1 solution from the MiSeq kit. For QIAseq, final library concentration (at a total volume of 600 µL) was 8 pM. The pool was sequenced using MiSeq v3 chemistry with 149 bp paired-end sequencing. For ‘custom primer panel + TruSeq DNA Nano’, the final library concentration was 10 pM. The pool was sequenced using MiSeq v3 chemistry with 181 bp paired-end sequencing. For KAPA HyperCap, the final library concentration was 8 pM. The pool was sequenced using MiSeq v3 chemistry with 76 bp paired-end sequencing.

### 4.5. Bioinformatics Analysis

Samples with fewer than 10,000 reads by at least one method were excluded from further analysis. Raw reads were filtered and trimmed with Trimmomatic (PE mode, ver. 0.39 USADELLAB) [24] with the following parameters: ILLUMINACLIP:TruSeq3-PE.fa:2:30:10 SLIDINGWINDOW:4:20 MINLEN:36 HEADCROP:30 (libraries prepared with the TruSeq DNA Nano Library Kit, QIAseq DIRECT SARS-CoV-2 Library Kit); and ILLUMINACLIP:TruSeq3-PE.fa:2:30:10 SLIDINGWINDOW:4:20 MINLEN:50 (KAPA HyperCap SARS-CoV-2 Library Kit). Trimmed reads were mapped to the Wuhan-Hu-1 SARS-CoV-2 reference genome (NCBI GenBank NC_045512.2) with bowtie2 (v.2.3.5.1) [25] in the local alignment mode.

All reads were then assigned to read groups by Picard Toolkit (ver. 2.27.4, Broad Institute) [26]. Variant calls were performed with GATK (ver. 4.2.6.1, Broad Institute) [27]. Variants with a quality score below 50 were excluded from further analysis. Consensus genomic sequences were created with bcftools (ver. 1.10.2) [28]. To identify genomic regions without proper coverage, we used a threshold of less than 5 reads per position.

For the comparison of SNPs identified through different approaches, the VCF Toolz Python package [29] was used. The visualization of results was performed with the R language [30] and ggplot2 package [31] scripts. Omicron variant sequences were uploaded to GISAID under the following IDs: EPI_ISL_14576113- EPI_ISL_14576150 and EPI_ISL_14701264. Delta variant sequences were uploaded under the following IDs: EPI_ISL_14840054-EPI_ISL_14840090 and EPI_ISL_14842468-EPI_ISL_14842470.

### 4.6. Statistical Analysis

Statistical analysis was performed with R statistical language (R Core Team, 2022). The statistical comparison of genomic coverage between the three sequencing approaches was conducted with the pairwise.t.test function (R language) with the p-adjustment method “FDR”.

## 5. Conclusions

When evaluating the results obtained, it can be concluded that all three approaches to creating libraries for SARS-CoV-2 sequencing can be used for research purposes. Each approach has its own advantages. In particular, with QIAseq DIRECT SARS-CoV-2, this is the speed of library preparation and a simple workflow. The capture-based KAPA HyperCap SARS-CoV-2 panel demonstrates the most accurate and complete coverage of the genome; however, in our experiments, we did not achieve a uniform distribution of reads across the samples. The developed custom panel has reliably proven itself in the genetic monitoring of various variants. There were no significant differences in genomic coverage or sequencing depth between the Delta and Omicron variants. Thus, at the moment, the custom panel is quite versatile for different variants of SARS-CoV-2. It also presents results comparable to other platforms, but with a rather simple workflow. Its predictable distribution of reads per sample is well suited for monitoring genetic variants as part of COVID-19 surveillance. In addition, the user panel remains compatible with new variants. Despite the introduction of a new set of Omicron mutations, the panel produces results of predictable quality. In other words, the amplicon approach is suitable for routine monitoring, while the hybridization capture approach is more valuable for scientific research and the discovery of new mutations and variants.

## Figures and Tables

**Table 1:** Workflow comparison for the different library kits used.

	QIAseq DIRECT SARS-CoV-2	KAPA HyperCap SARS-CoV-2	Custom Primer Panel + TruSeq DNA Nano
Fragmentation	N	Y	N
Reverse transcription	Y	Y	Y
Second-strand synthesis	N	Y	N
PCR	21 + 6 cycles	8 + 18 cycles	35 + 8 cycles
Hybridization	N	1–16 h	N
Estimated time for library construction *	4 h	7 h **	6 h ***
Max Ct ****	20	23	23
Input RNA amount, ng *	Not specified	10–100	<10

* according to the manufacturer’s protocol, ** not including time for hybridization, *** not including time for genome enrichment, **** based on our observations.

## Data Availability

Data sharing not applicable.

## Supplementary Materials

The following supporting information can be downloaded at: https://www.mdpi.com/article/10.3390/ijms24032374/s1, Supplementary S1: Table S1. Assembly statistics for TruSeq DNA Nano Library Kit with custom primer panel, Table S2. Assembly statistics for KAPA HyperCap SARS-CoV-2 kit, Table S3. Assembly statistics for QIAseq DIRECT SARS-CoV-2 kit. Supplementary S2: Table S4. Number of raw reads for each of three approaches, Table S5. Percent of remaining reads after trimming for each of three approaches, Table S6. Percent of genomic coverage for each of three approaches, Table S7. Number of variants for each of three approaches. Supplementary S3: Table S8. SNP profile for each sample (TruSeq DNA Nano Library Kit with custom primer panel), Table S9. SNP profile for each sample (KAPA HyperCap SARS-CoV-2 kit), Table S10. SNP profile for each sample (QIAseq DIRECT SARS-CoV-2 kit).
