# Supplementary material for: Comparative Analysis of Library Preparation Approaches for SARS-CoV-2 Genome Sequencing on the Illumina MiSeq Platform
Source: Int J Mol Sci. 2023 Jan 25;24(3):2374. doi: 10.3390/ijms24032374 (PMC9916928; doi:10.3390/ijms24032374)
Supplement: Supplementary file 1 [file ijms-24-02374-s001.zip › Supplementary 2.pdf]

Table S4. Number of raw reads for each of three approaches

	KAPA HyperCap SARS-CoV-2	QIAseq DIRECT SARS-CoV-2	TruSeq DNA Nano with custom primer panel
Minimum number of reads	12813	10794	179646
Maximum number of reads	3402936	707446	359999
Mean	480964	161294	254036
Median	134720	100524	258838

Table S5. Percent of remaining reads after trimming for each of three approaches

	KAPA HyperCap SARS-CoV-2	QIAseq DIRECT SARS-CoV-2	TruSeq DNA Nano with custom primer panel
Min	12.78	5.13	91.98
Max	83.45	81.85	95.36
Mean	39.21	55.83	93.79
Median	34.42	59.82	93.81

Table S6. Percent of genomic coverage for each of three approaches

	KAPA HyperCap SARS-CoV-2	QIAseq DIRECT SARS-CoV-2	TruSeq DNA Nano with custom primer panel
Min	99.70	50.71	93.90
Max	99.99	97.64	99.02
Mean	99.86	88.00	97.98
Median	99.87	92.12	98.25

Table S7. Number of variants for each of three approaches

	KAPA HyperCap SARS-CoV-2	QIAseq DIRECT SARS-CoV-2	TruSeq DNA Nano with custom primer panel
Min	65	31	64
Max	78	69	76
Mean	72.28	56.65	71
Median	73.00	58.50	71
